# Species-Dependent Antibacterial Responses and Bioactivity Patterns Associated with Declared Botanical Type in Honey: An Integrated Biochemical and Microbiological Analysis

**DOI:** 10.3390/molecules31173048

**Published:** 2026-08-30

**Authors:** Filip Lewandowski, Rafał Hrynkiewicz, Dominika Bębnowska, Joanna Ziętara-Wysocka, Paulina Niedźwiedzka-Rystwej

**Affiliations:** 1 Department of Microbiology and Immunology, Institute of Biology, University of Szczecin, Felczaka 3c, 71-412 Szczecin, Poland; filip.lewandowski@usz.edu.pl (F.L.); rafal.hrynkiewicz@usz.edu.pl (R.H.); dominika.bebnowska@usz.edu.pl (D.B.); joanna.zietara-wysocka@phd.usz.edu.pl (J.Z.-W.); 2 Centre for Experimental Immunology and Immunobiology in Infectious Diseases and Cancer, University of Szczecin, Felczaka 3c, 71-412 Szczecin, Poland; 3 Doctoral School, University of Szczecin, Mickiewicza 18, 70-384 Szczecin, Poland

**Keywords:** honey, bacterial susceptibility, botanical origin, antimicrobial activity, MIC, MBC, bioactivity fingerprint, multivariate analysis

## Abstract

Honey antibacterial activity reflects both the properties of the honey matrix and the susceptibility of the target microorganism, but the consistency with which these sources of variation are reflected across antimicrobial endpoints remains insufficiently characterised. This study investigated whether species-dependent differences in antibacterial responses were detected more consistently than differences among declared botanical honey types and whether the integration of antimicrobial and biochemical traits could reveal botanical-type-associated bioactivity patterns not apparent from individual endpoints. Thirty-seven honey samples representing seven declared botanical types from Western Pomerania, Poland, were analysed for total phenolic content (TPC), ferric-reducing antioxidant power (FRAP), operational minimum inhibitory concentration producing ≥90% growth inhibition (MIC90), and minimum bactericidal concentration (MBC) against *Staphylococcus aureus*, *Staphylococcus epidermidis*, *Escherichia coli*, *Pseudomonas aeruginosa*, and *Proteus vulgaris*. Buckwheat honeys showed the highest observed TPC and FRAP values among the analysed samples. Species-dependent differences in MIC90 and MBC were detected more consistently than differences among declared botanical types, whereas significant botanical-type effects for individual antimicrobial endpoints were observed only for *S. aureus*. *P. vulgaris* was generally the most susceptible species, although susceptibility patterns differed between inhibitory and bactericidal endpoints. Associations between biochemical parameters and antimicrobial activity were limited and directionally inconsistent. In contrast, the integration of five MIC90 variables, five MBC variables, FRAP activity, and TPC revealed significant botanical-type-associated structuring of the overall bioactivity profile. This association remained significant after the exclusion of botanical types represented by single samples (pseudo-F = 2.66, R^2^ = 0.283, *p* = 0.0001), while multivariate dispersion no longer differed significantly among groups. The resulting bioactivity fingerprint should be regarded as a descriptive multidimensional representation of the investigated samples rather than as a validated classification or prediction tool. Within the present dataset, combined species-resolved microbiological and biochemical profiling provided a more comprehensive description of honey bioactivity than individual antimicrobial or biochemical endpoints considered separately.

## 1. Introduction

Honey is a heterogeneous antimicrobial food matrix whose biological activity cannot be adequately described by a single physicochemical or chemical characteristic. Its antibacterial effects arise from the combined action of high osmolarity, low pH, hydrogen peroxide generation, antimicrobial peptides, phenolic constituents and, in some honeys, specific non-peroxide factors such as methylglyoxal [1,2]. The relative contribution of these mechanisms varies substantially among honey samples, leading to considerable differences in antibacterial potency even among honeys assigned to the same botanical category [3,4,5]. Consequently, botanical origin, although closely associated with honey composition and widely used to categorise its functional properties, may not provide a direct or sufficient proxy for antibacterial performance. 

A second source of variability is inherent to the microorganism exposed to honey. The antibacterial phenotype observed experimentally is therefore not solely a property of the honey, but the outcome of an interaction between the antimicrobial characteristics of the matrix and the susceptibility of the target bacterium. This distinction has been recognised in food microbiology for decades. Mundo et al. demonstrated that bacteria were not uniformly affected by the same honey samples and that antimicrobial activity varied markedly among microorganisms and even among strains of *Staphylococcus aureus*; importantly, the observed activity could not be consistently attributed to a specific floral source or geographical origin [6]. Subsequent studies have likewise reported substantial microorganism-dependent variation in antibacterial responses to different honeys [7,8,9,10]. These observations challenge the assumption that a botanical honey type can be assigned a general antibacterial potency independently of the microorganism used as the test system. 

Botanical origin nevertheless leaves a measurable biochemical signature. Phenolic composition and antioxidant capacity differ among honey varieties, and Polish varietal honeys provide a particularly clear example of this relationship. Jaśkiewicz et al. found substantial botanical-type-associated differences in phenolic composition and antioxidant activity among 84 Polish honeys, but neither clustering nor principal component analysis produced complete separation of all varieties, which the authors attributed in part to pronounced within-variety heterogeneity [11]. This is particularly relevant when biological activity is considered: biochemical characteristics associated with botanical origin do not necessarily translate into proportional changes in antibacterial activity. For example, relationships among colour, phenolic content, antioxidant capacity and MIC/MBC have been demonstrated in selected honey–bacterium systems, including Spanish honeys tested against *S. epidermidis* [12], whereas broader studies involving several honey types and microorganisms have revealed substantially more heterogeneous relationships [8,9]. Thus, individual biochemical indicators such as total phenolic content or reducing capacity may describe selected dimensions of honey bioactivity but cannot be assumed to predict its antibacterial phenotype across bacterial species. 

This apparent disconnect between botanical classification, biochemical composition and microorganism-specific antibacterial activity suggests that honey bioactivity may be better conceptualised as a multidimensional phenotype rather than as a set of independent functional traits. Multivariate approaches have already demonstrated that information distributed across several physicochemical, compositional or bioactive variables can reveal botanical or geographical structure that is not equally apparent from individual measurements. In Brazilian honeys, the integration of bioactive, antioxidant and antimicrobial characteristics was used to identify features differentiating samples according to botanical and geographical origin [13]. More recently, a polyparametric chemoinformatic approach combining multiple honey characteristics improved discrimination according to botanical origin compared with individual parameter sets [14]. Conversely, the incomplete separation observed among Polish varietal honeys based on phenolic and antioxidant parameters alone illustrates how substantial within-type heterogeneity may limit classification based on a restricted subset of traits [11]. Together, these findings provide a rationale for extending multivariate profiling from compositional characterisation towards functional bioactivity fingerprinting, in which biochemical properties are integrated with species-resolved antibacterial responses. 

Accordingly, the present study was designed to test two interrelated hypotheses: (1) species-dependent differences in antibacterial responses would be detected more consistently across MIC90 and MBC endpoints than differences among declared botanical types, without implying a direct quantitative comparison of the relative effect sizes of these factors; and (2) the integration of species-specific antibacterial responses with biochemical characteristics would reveal botanical-type-associated patterns of bioactivity that were not consistently detectable when individual parameters were analysed separately. To test these hypotheses, 37 honey samples representing seven declared botanical types from Western Pomerania, Poland, were characterised for operational MIC90 and MBC against *Staphylococcus aureus*, *Staphylococcus epidermidis*, *Escherichia coli*, *Pseudomonas aeruginosa*, and *Proteus vulgaris*, together with TPC and FRAP antioxidant activity. Univariate and repeated-measures analyses were complemented by multivariate modelling and ordination to characterise microorganism-dependent susceptibility and botanical-type-associated variation. Finally, species-specific antimicrobial and biochemical variables were integrated to derive a descriptive bioactivity fingerprint representing the combined inhibitory, bactericidal, and biochemical characteristics of the investigated honey samples.

## 2. Results

### 2.1. Dataset Characteristics

The analysis included 37 honey samples representing seven declared botanical types: phacelia (n = 1), buckwheat (n = 2), linden (n = 4), rapeseed (n = 12), honeydew (n = 1), multifloral (n = 15), and heather (n = 2). For each sample, the operational minimum inhibitory concentration producing ≥90% growth inhibition (MIC90) and minimum bactericidal concentration (MBC) were determined against five bacterial species: *Escherichia coli*, *Pseudomonas aeruginosa*, *Proteus vulgaris*, *Staphylococcus aureus*, and *Staphylococcus epidermidis*, resulting in 185 sample–bacterial species combinations. Because phacelia and honeydew were each represented by a single sample and the remaining groups were also unequal in size, results for sparsely represented botanical types were interpreted descriptively and the influence of singleton groups was additionally examined in the multivariate sensitivity analysis.

### 2.2. Antioxidant Activity and Total Phenolic Content

**Figure 1:**
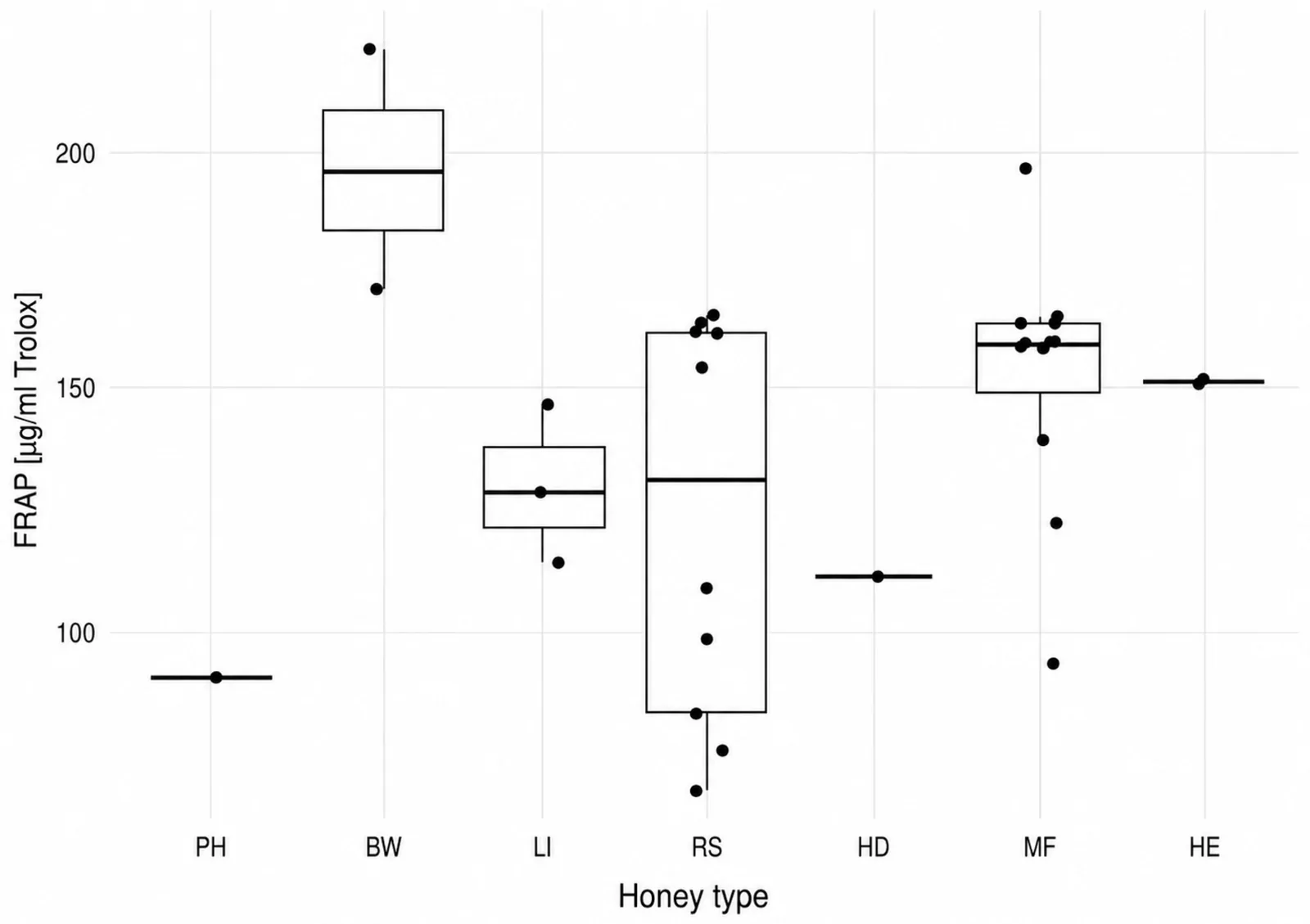
**FRAP antioxidant activity of honey samples according to declared botanical type.** Individual points represent independent honey samples. Boxplots show the median and interquartile range. FRAP activity is expressed as µg/mL Trolox equivalents (TE). PH, phacelia; BW, buckwheat; LI, linden; RS, rapeseed; HD, honeydew; MF, multifloral; HE, heather.

FRAP antioxidant activity varied among the analysed honey samples and declared botanical groups. Among the botanical types represented by more than one sample, buckwheat honeys showed the highest observed FRAP values (mean and median: 195.90 µg/mL TE; range: 172.23–219.57 µg/mL TE). Heather honeys showed FRAP values of 150.90–151.57 µg/mL TE, whereas multifloral honeys displayed greater within-group variability (65.57–197.23 µg/mL TE). The single honeydew and phacelia samples showed FRAP values of 112.57 and 92.57 µg/mL TE, respectively; these individual observations were treated descriptively and should not be interpreted as representative estimates for the corresponding botanical types. The distribution of FRAP antioxidant activity across declared botanical types is shown in Figure 1.

**Figure 2:**
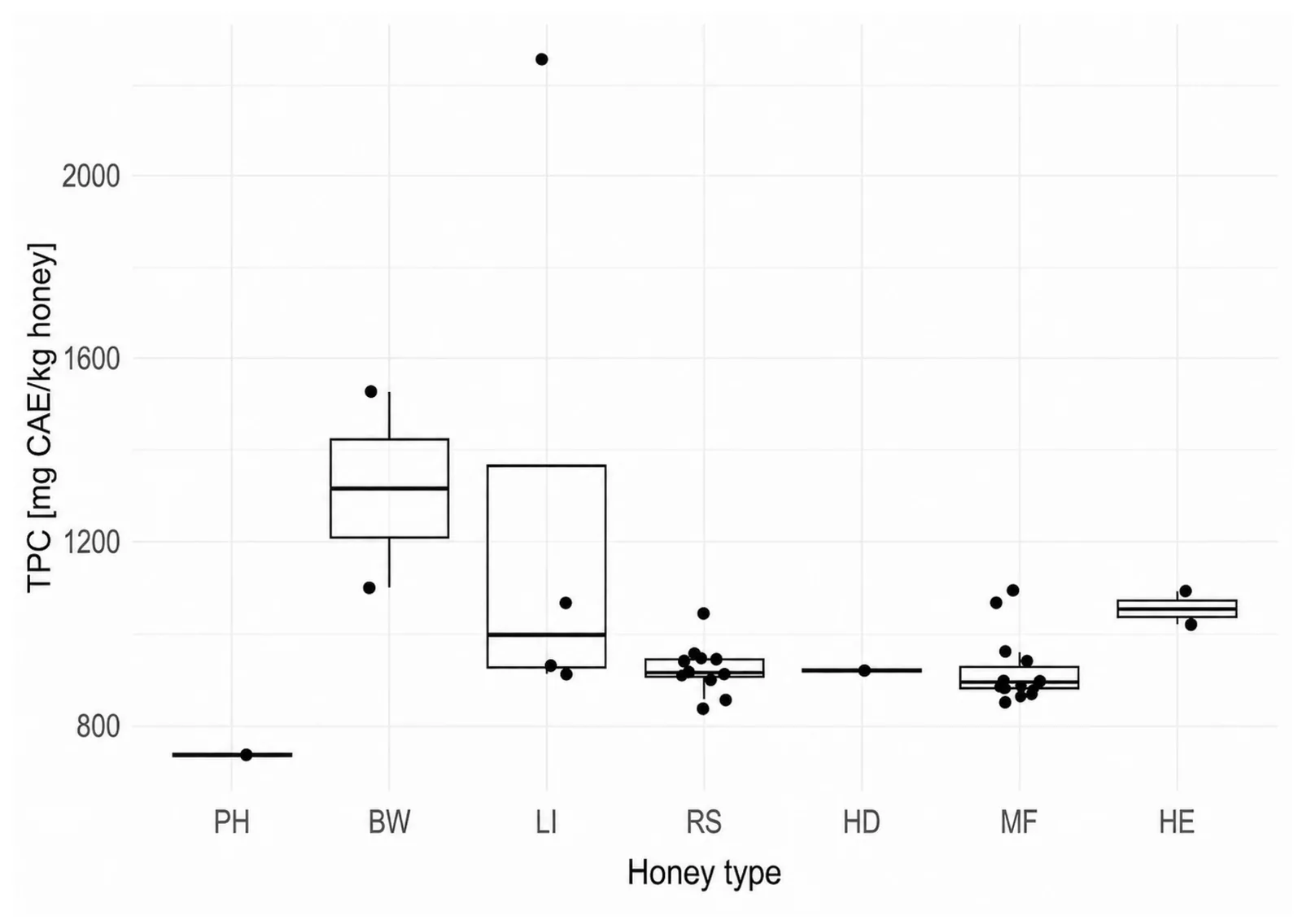
**Total phenolic content of honey samples according to declared botanical type.** Individual points represent independent honey samples. Boxplots show the median and interquartile range. Total phenolic content is expressed as milligrams of chlorogenic acid equivalents per kilogram of honey (mg CAE/kg honey). PH, phacelia; BW, buckwheat; LI, linden; RS, rapeseed; HD, honeydew; MF, multifloral; HE, heather.

Total phenolic content (TPC) also varied among samples and declared botanical groups. Buckwheat honeys showed the highest observed group-level TPC among the analysed samples (mean and median: 1320.82 mg CAE/kg honey; range: 1104.32–1537.32 mg CAE/kg honey). Linden honeys showed substantial within-group variability, with values ranging from 926.56 to 2207.32 mg CAE/kg honey. Heather, rapeseed, and multifloral honeys showed intermediate values, whereas the single honeydew and phacelia samples yielded TPC values of 935.67 and 748.80 mg CAE/kg honey, respectively. As for FRAP, values obtained for botanical types represented by single samples were considered descriptive only. The distribution of TPC across declared botanical types is shown in Figure 2.

Numerical summaries of FRAP, TPC, MIC90, and MBC values according to declared botanical type are provided in Table S1.

**Figure 3:**
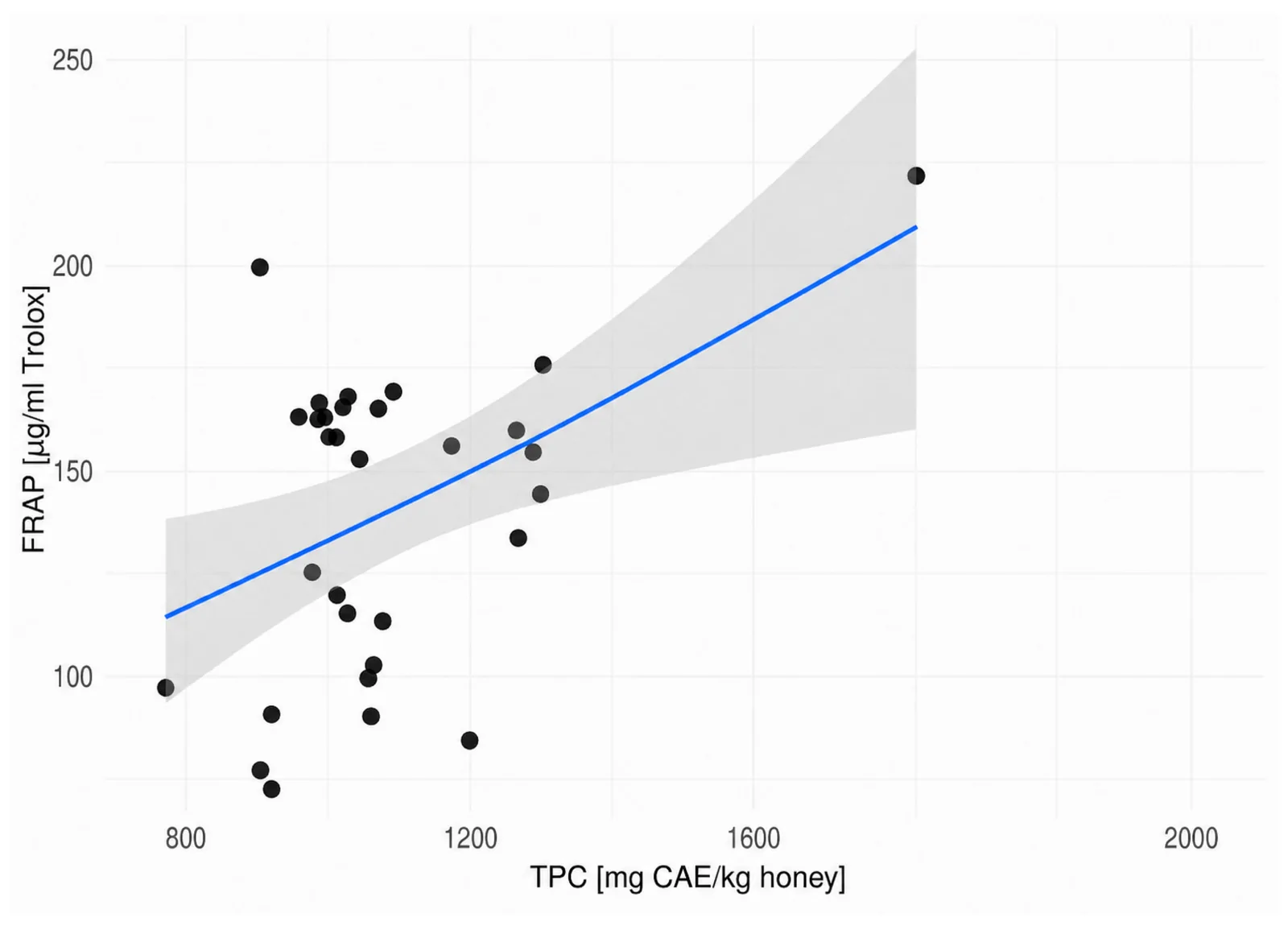
**Relationship between total phenolic content and FRAP antioxidant activity across individual honey samples.** Each point represents an independent honey sample. Total phenolic content is expressed as mg CAE/kg honey and FRAP antioxidant activity as µg/mL Trolox equivalents (TE). The fitted line and shaded 95% confidence interval are shown for visualisation of the overall trend.

The relationship between total phenolic content and FRAP antioxidant activity across individual honey samples is shown in Figure 3.

### 2.3. Variation in Antimicrobial Activity Among Declared Botanical Types

**Figure 4:**
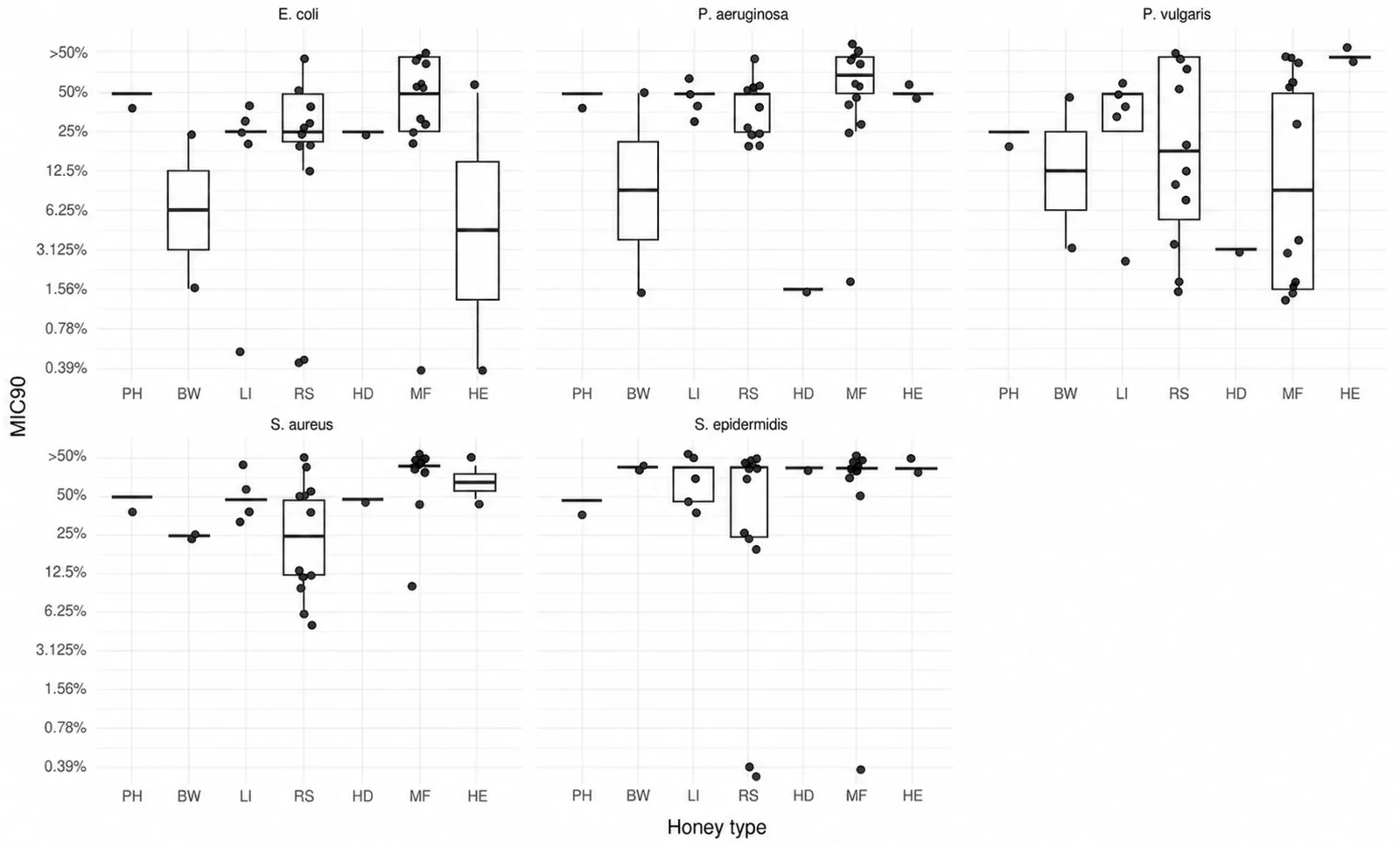
**Operational minimum inhibitory concentration producing ≥90% growth inhibition (MIC90) of honey samples against five bacterial species according to declared botanical type.** MIC90 values are shown for *Escherichia coli*, *Pseudomonas aeruginosa*, *Proteus vulgaris*, *Staphylococcus aureus*, and *Staphylococcus epidermidis*. Individual points represent independent honey samples and boxplots show the median and interquartile range. Lower MIC90 values indicate stronger inhibitory activity. PH, phacelia; BW, buckwheat; LI, linden; RS, rapeseed; HD, honeydew; MF, multifloral; HE, heather.

**Figure 5:**
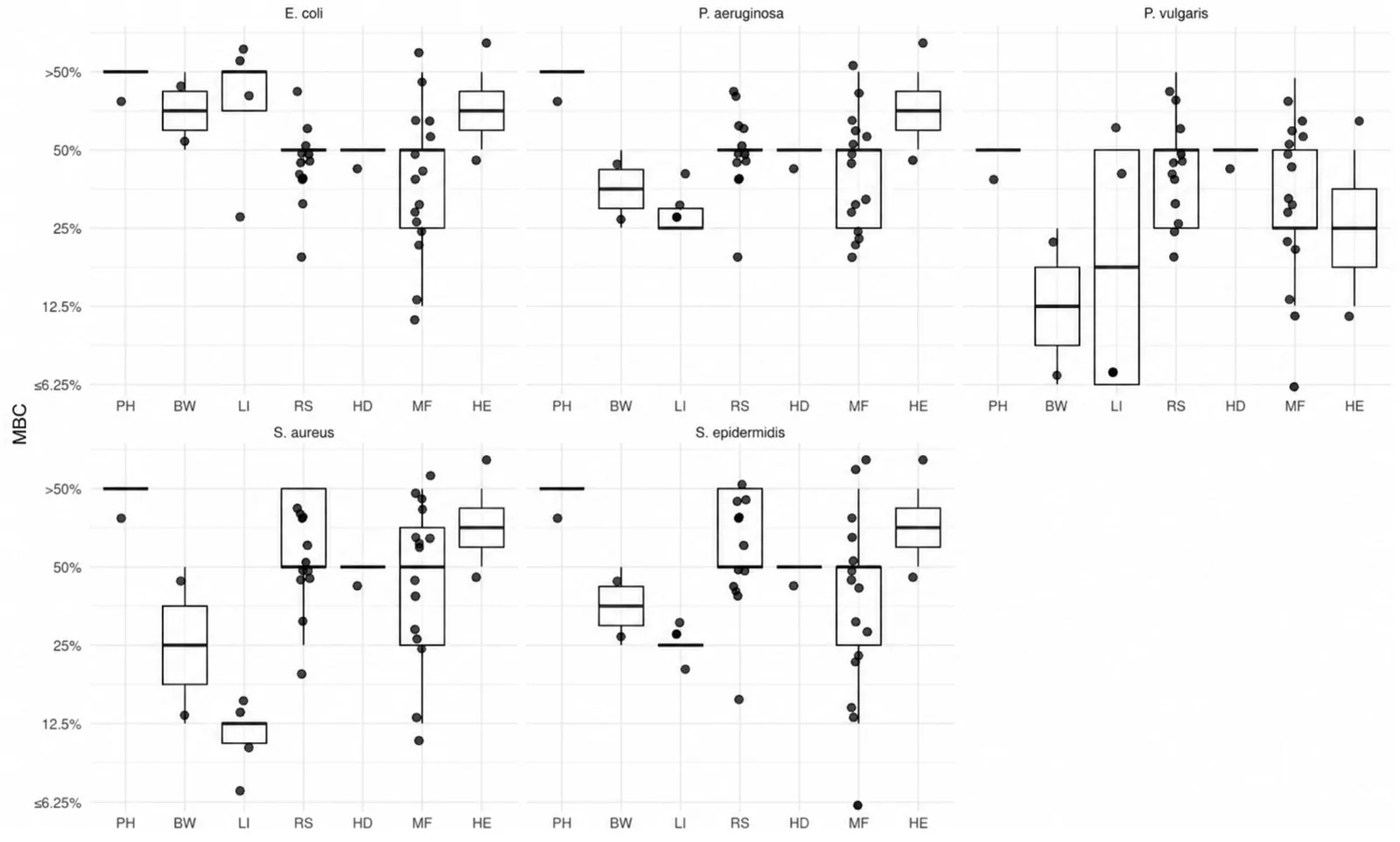
**Minimum bactericidal concentration (MBC) of honey samples against five bacterial species according to declared botanical type.** MBC values are shown for *Escherichia coli*, *Pseudomonas aeruginosa*, *Proteus vulgaris*, *Staphylococcus aureus*, and *Staphylococcus epidermidis*. Individual points represent independent honey samples and boxplots show the median and interquartile range. Lower MBC values indicate stronger bactericidal activity under the operational MBC definition used in this study. PH, phacelia; BW, buckwheat; LI, linden; RS, rapeseed; HD, honeydew; MF, multifloral; HE, heather.

MIC90 and MBC values showed considerable variability both among declared botanical types and within individual botanical groups, particularly among multifloral honeys. In species-specific univariate analyses, declared botanical type was significantly associated with antimicrobial activity only against *Staphylococcus aureus*. For MIC90, the Kruskal–Wallis test yielded H = 16.00, df = 6, and *p* = 0.0137, whereas for MBC the corresponding values were H = 13.85, df = 6, and *p* = 0.0313. No statistically significant differences among declared botanical types were observed for the remaining bacterial species. These results indicate that the association between declared botanical type and antibacterial activity was dependent on both the bacterial species and the antimicrobial endpoint rather than representing a uniform effect across all tested microorganisms. The distributions of MIC90 and MBC values across declared botanical types and bacterial species are shown in Figure 4 and Figure 5, respectively.

Post hoc analysis for *S. aureus* showed that rapeseed honeys exhibited stronger inhibitory activity than multifloral honeys. The median MIC90 code was 7 for rapeseed honey, corresponding to 25% (*v*/*v*), compared with a median code of 9 for multifloral honey, corresponding to >50% (*v*/*v*) (Dunn’s test with Benjamini–Hochberg correction, p_BH = 0.0058). Thus, the median rapeseed sample reached the operational MIC90 endpoint at 25% honey, whereas the median multifloral sample did not reach the ≥90% inhibition threshold even at the highest tested concentration of 50%. This difference therefore represented a substantial separation within the tested concentration range rather than a statistical difference in ordinal codes alone.

For bactericidal activity against *S. aureus*, linden honeys showed greater activity than rapeseed honeys. The median MBC code was 2 for linden honey, corresponding to 12.5% (*v*/*v*), compared with a median code of 4 for rapeseed honey, corresponding to 50% (*v*/*v*) (p_BH = 0.0320). This corresponds to a fourfold difference in the tested honey concentration required to reach the operational MBC endpoint. Nevertheless, this finding should be interpreted within the operational conditions of the MBC assay and in view of the unequal botanical-group sizes. None of the remaining pairwise comparisons remained statistically significant after correction for multiple comparisons.

When the five species-specific MBC variables were analysed jointly, declared botanical type was significantly associated with the multivariate bactericidal activity profile (pseudo-F = 2.27, R^2^ = 0.312, *p* = 0.0104). However, multivariate dispersion differed significantly among botanical groups (betadisper *p* = 0.0300). Accordingly, this PERMANOVA result cannot be attributed exclusively to differences in group centroids and should be interpreted cautiously because differences in within-group variability may also have contributed to the detected effect.

In contrast, no significant association between declared botanical type and the multivariate MIC90 profile was observed (pseudo-F = 1.03, R^2^ = 0.171, *p* = 0.4261), while multivariate dispersion was homogeneous among groups (betadisper *p* = 0.3005).

### 2.4. Differences in Bacterial Species Susceptibility

Susceptibility to the antibacterial effects of honey differed significantly among the five bacterial species tested. For MBC, the Friedman test indicated significant differences among species (χ^2^ = 12.49, df = 4, *p* = 0.0141). The lowest mean MBC code, corresponding to the greatest susceptibility to bactericidal activity, was observed for *Proteus vulgaris* (3.32), followed by *Staphylococcus aureus* (3.73), *Staphylococcus epidermidis* (3.78), *Pseudomonas aeruginosa* (3.84) and *Escherichia coli* (3.92).

After Benjamini–Hochberg correction, *P. vulgaris* showed significantly lower MBC codes than *E. coli*, *P. aeruginosa*, and *S. epidermidis* (p_BH = 0.0291 for each comparison). No other pairwise differences in MBC remained statistically significant after correction for multiple comparisons. Differences among bacterial species were more pronounced for MIC90 (χ^2^ = 32.85, df = 4, *p* < 0.001). The lowest mean MIC90 codes were observed for *P. vulgaris* (6.35) and *E. coli* (6.65), whereas higher mean values were recorded for *P. aeruginosa* (7.62), *S. aureus* (7.92), and *S. epidermidis* (8.08). After correction for multiple comparisons, MIC90 codes for *P. vulgaris* were significantly lower than those for *S. aureus* (p_BH = 0.0140), *S. epidermidis* (p_BH = 0.0190), and *P. aeruginosa* (p_BH = 0.0316). *E. coli* also showed significantly lower MIC90 codes than *S. aureus* and *S. epidermidis* (p_BH = 0.0144 for both comparisons), as well as *P. aeruginosa* (p_BH = 0.0401).

Overall, species-dependent differences were detected across a greater number of pairwise comparisons than differences among declared botanical types. This pattern indicates that microorganism-specific susceptibility was more consistently reflected in the antimicrobial endpoints within the present dataset. However, because bacterial species and declared botanical type were evaluated using different statistical frameworks, these results should not be interpreted as a direct quantitative comparison of their relative effect sizes.

### 2.5. Relationships Between Biochemical and Antimicrobial Parameters

Exploratory Spearman correlation analysis identified three nominal associations between biochemical parameters and species-specific antimicrobial endpoints. TPC was negatively correlated with the MIC90 code for *Escherichia coli* (ρ = −0.39; nominal *p* = 0.0195), indicating that higher TPC values were associated with lower MIC90 codes and therefore stronger inhibitory activity. In contrast, TPC was positively correlated with the MBC code for the same bacterial species (ρ = 0.36; nominal *p* = 0.0298), indicating an association with weaker bactericidal activity. FRAP antioxidant activity was positively correlated with the MIC90 code for Staphylococcus epidermidis (ρ = 0.35; nominal *p* = 0.0431), corresponding to higher MIC90 codes at higher FRAP values.

However, none of these associations remained statistically significant after Benjamini–Hochberg FDR correction for the 20 biochemical–antimicrobial correlations evaluated (all p_BH ≥ 0.05). The nominal associations should therefore be regarded as exploratory observations rather than confirmatory evidence of relationships between TPC or FRAP and antibacterial activity.

**Figure 6:**
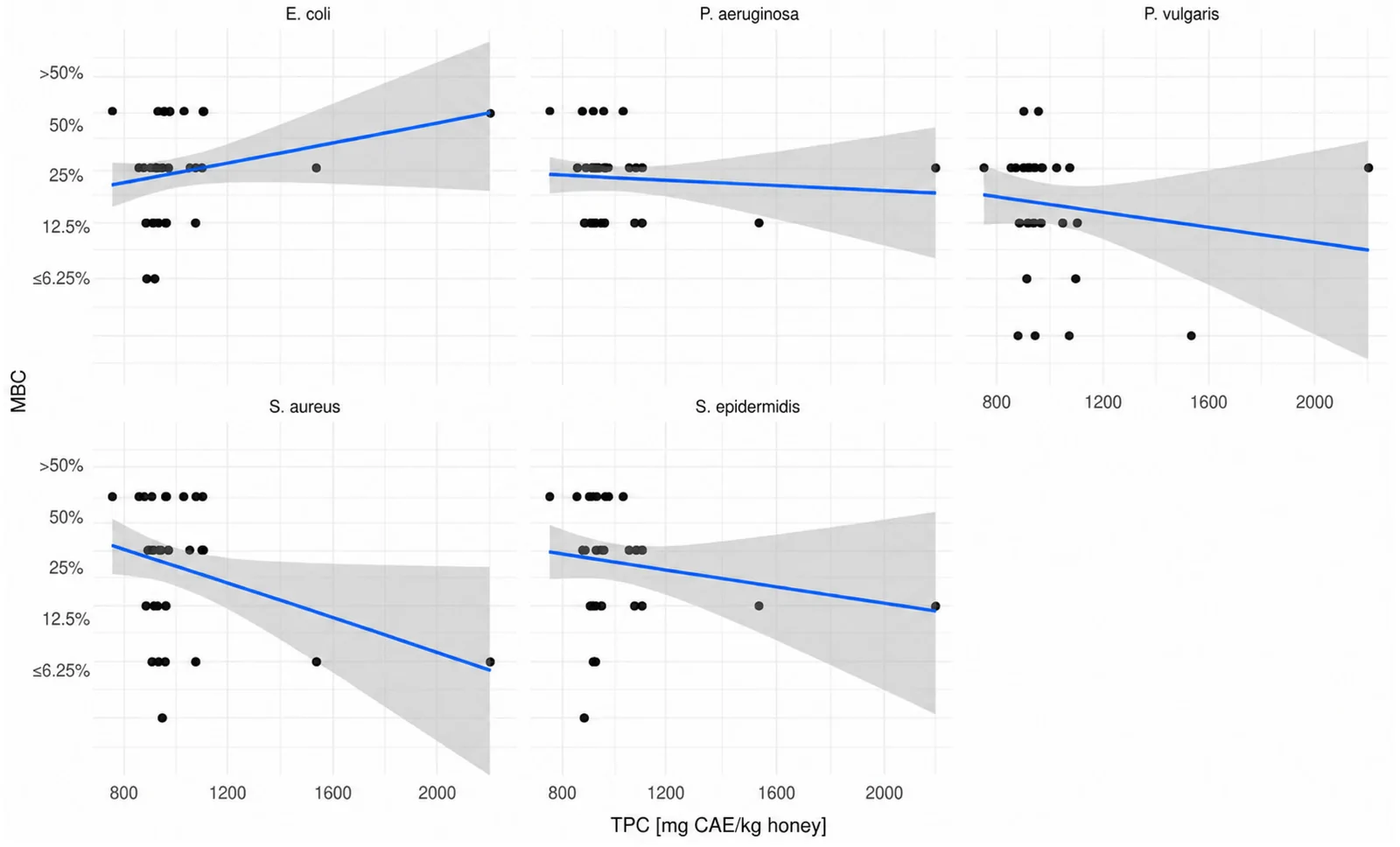
**Relationships between total phenolic content and minimum bactericidal concentration (MBC) across individual honey samples.** Relationships are shown separately for *Escherichia coli*, *Pseudomonas aeruginosa*, *Proteus vulgaris*, *Staphylococcus aureus*, and *Staphylococcus epidermidis*. Lower MBC values indicate stronger bactericidal activity. Lines and shaded 95% confidence intervals are included to visualise the direction of the relationships; inferential associations were evaluated using Spearman’s rank correlation analysis.

**Figure 7:**
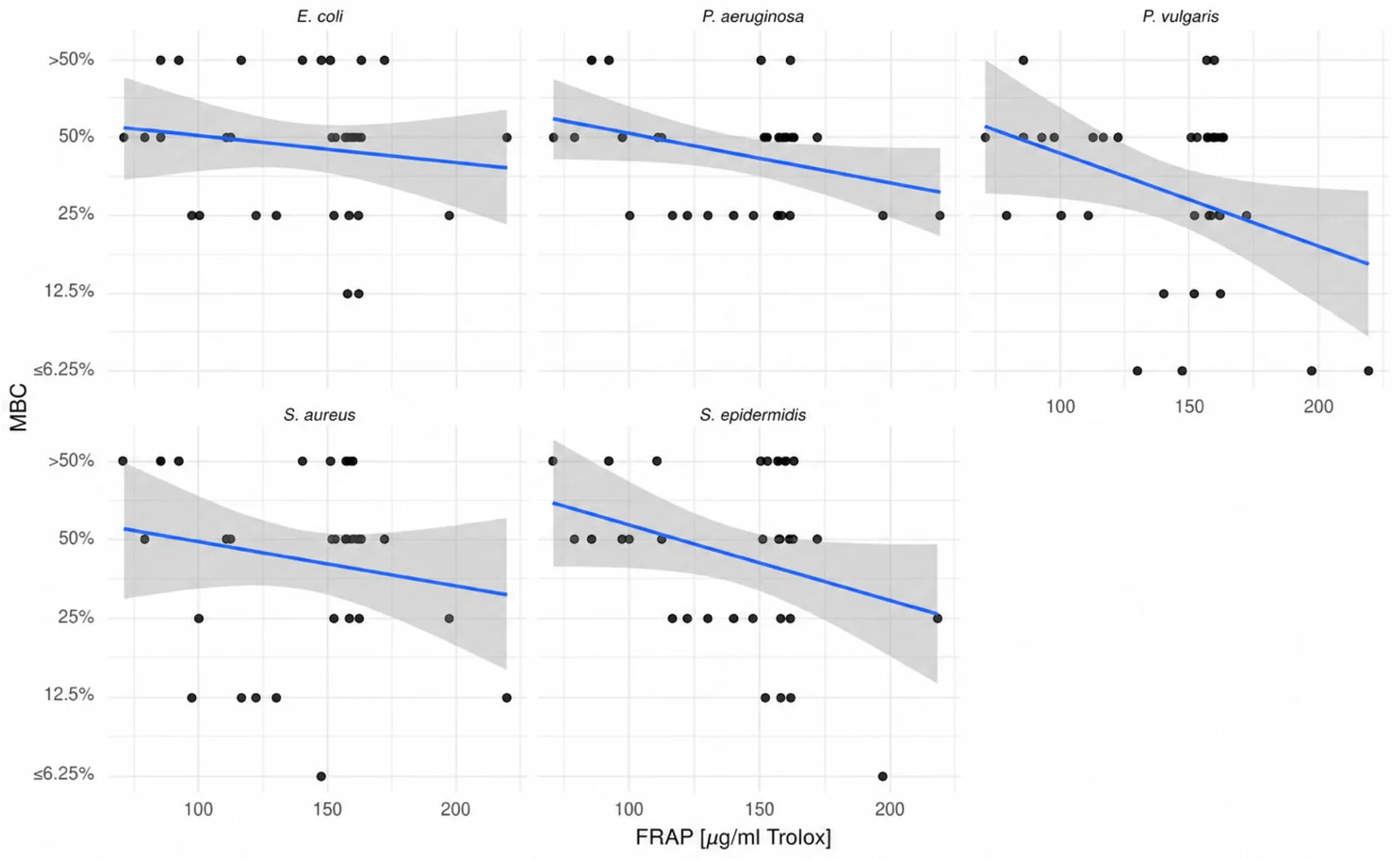
**Relationships between FRAP antioxidant activity and minimum bactericidal concentration (MBC) across individual honey samples.** Relationships are shown separately for *Escherichia coli*, *Pseudomonas aeruginosa*, *Proteus vulgaris*, *Staphylococcus aureus*, and *Staphylococcus epidermidis*. FRAP activity is expressed as µg/mL Trolox equivalents (TE), and lower MBC values indicate stronger bactericidal activity. Lines and shaded 95% confidence intervals are included to visualise the direction of the relationships; inferential associations were evaluated using Spearman’s rank correlation analysis.

The differing directions of the observed associations between inhibitory and bactericidal endpoints further indicate that neither TPC nor FRAP can be considered a direct predictor of honey antibacterial activity across the bacterial species investigated. Species-specific relationships between TPC and MBC and between FRAP antioxidant activity and MBC are visualised in Figure 6 and Figure 7, respectively.

### 2.6. Integrated Bioactivity Profile of Declared Botanical Honey Types

PERMANOVA incorporating the five species-specific MIC90 variables, five MBC variables, FRAP antioxidant activity, and TPC revealed a significant association between declared botanical type and the integrated bioactivity profile in the complete dataset (pseudo-F = 2.18, R^2^ = 0.326, *p* = 0.0005). However, multivariate dispersion also differed significantly among botanical groups (betadisper: F = 3.04, *p* = 0.0256). Therefore, the significant PERMANOVA result for the complete dataset cannot be attributed exclusively to differences in group centroids, because differences in within-group dispersion may also have contributed to the observed multivariate separation.

To evaluate the robustness of the botanical-type-associated pattern, the analysis was repeated after exclusion of the phacelia and honeydew groups, which were each represented by a single sample. In this sensitivity analysis, the association between declared botanical type and the integrated bioactivity profile remained significant (pseudo-F = 2.66, R^2^ = 0.283, *p* = 0.0001), whereas no significant differences in multivariate dispersion were detected (F = 0.68, *p* = 0.6029). This sensitivity analysis therefore provides a more reliable basis for interpreting the botanical-type-associated multivariate structure because the significant PERMANOVA result was not accompanied by detectable heterogeneity of within-group dispersion.

Pairwise PERMANOVA comparisons were performed between declared botanical types represented by more than one sample. After Benjamini–Hochberg correction, significant differences in the integrated bioactivity profile were observed between rapeseed and multifloral honeys (pseudo-F = 3.16, R^2^ = 0.121, p_BH = 0.0100), rapeseed and linden honeys (pseudo-F = 3.60, R^2^ = 0.217, p_BH = 0.0100), multifloral and buckwheat honeys (pseudo-F = 3.29, R^2^ = 0.202, p_BH = 0.0270), and rapeseed and buckwheat honeys (pseudo-F = 3.99, R^2^ = 0.249, p_BH = 0.0293). The comparison between multifloral and linden honeys did not remain statistically significant after correction (p_BH = 0.0760).

The envfit analysis indicated that the complete-dataset ordination configuration was most strongly associated with MBC against *Proteus vulgaris* (R^2^ = 0.688), *Staphylococcus epidermidis* (R^2^ = 0.679), and *Staphylococcus aureus* (R^2^ = 0.656); all three associations remained significant after Benjamini–Hochberg correction (p_BH < 0.001). Significant fitted associations were also observed for MIC90 against *Escherichia coli* (R^2^ = 0.546), MBC against *E. coli* (R^2^ = 0.494), MBC against *Pseudomonas aeruginosa* (R^2^ = 0.470), TPC (R^2^ = 0.438), and FRAP antioxidant activity (R^2^ = 0.394).

**Figure 8:**
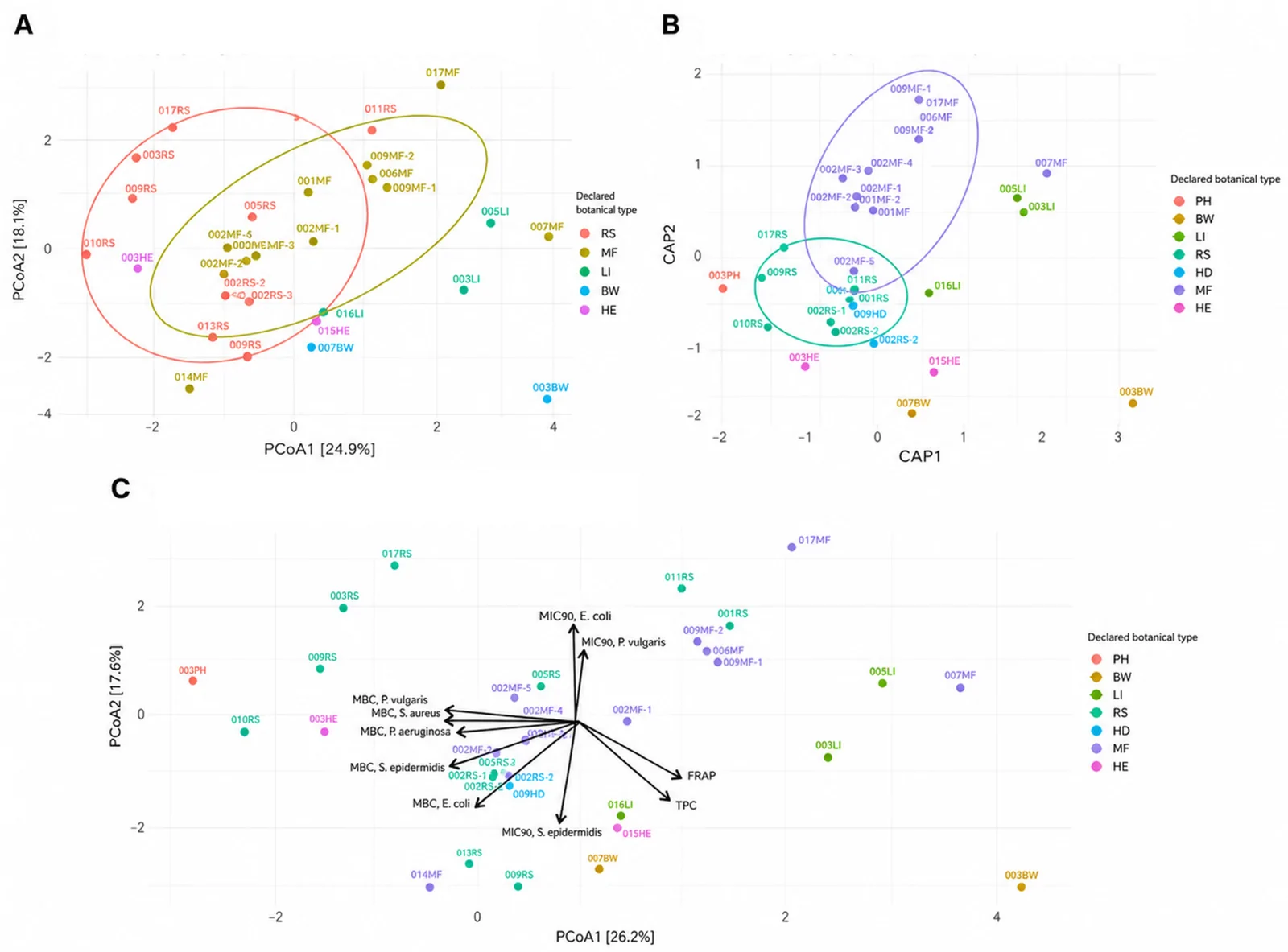
**Ordination analysis of the integrated bioactivity profiles of honey samples according to declared botanical type.** (**A**) Principal coordinates analysis (PCoA) performed after exclusion of declared botanical types represented by a single sample. Ellipses illustrate the distribution of the major botanical groups in ordination space; the corresponding PERMANOVA showed a significant association between declared botanical type and the integrated bioactivity profile (pseudo-F = 2.66, R^2^ = 0.283, *p* = 0.0001). (**B**) Constrained analysis of principal coordinates/distance-based redundancy analysis (CAP/dbRDA) of the integrated bioactivity profile using declared botanical type as the constraining factor. Ellipses illustrate the grouping of samples according to declared botanical type in the constrained ordination space. (**C**) PCoA of the complete dataset based on the integrated profile comprising five MIC90 variables, five MBC variables, FRAP antioxidant activity, and total phenolic content (TPC). Fitted *envfit* vectors indicate the direction and strength of associations between individual variables and the ordination configuration. PH, phacelia; BW, buckwheat; LI, linden; RS, rapeseed; HD, honeydew; MF, multifloral; HE, heather.

PCoA performed after exclusion of botanical types represented by a single sample visualised the botanical-type-associated structure corresponding to the sensitivity PERMANOVA analysis (Figure 8A). Complementary constrained ordination using CAP/dbRDA further illustrated the structure of the integrated bioactivity profiles according to declared botanical type (Figure 8B). PCoA of the complete dataset showed the overall multivariate configuration and the relationships between individual samples and fitted antimicrobial and biochemical variables (Figure 8C). These ordination methods were used for visualisation of the multivariate structure and should not be interpreted as independent confirmatory tests of botanical classification.

Following ordinal rescaling of MIC90 and MBC codes to a common 0–1 activity scale, descriptive differences in global antimicrobial activity indices were observed among declared botanical types. Buckwheat honeys showed the highest median global MIC90 activity index (0.312), whereas linden honeys showed the highest median global MBC activity index (0.500). Multifloral honeys had an intermediate median global MBC activity index (0.350) and showed considerable between-sample variability. Rapeseed honeys displayed comparatively uniform profiles, with median global MIC90 and MBC activity indices of 0.225 for both endpoints. Heather honeys showed median global MIC90 and MBC activity indices of 0.150 and 0.200, respectively.

**Figure 9:**
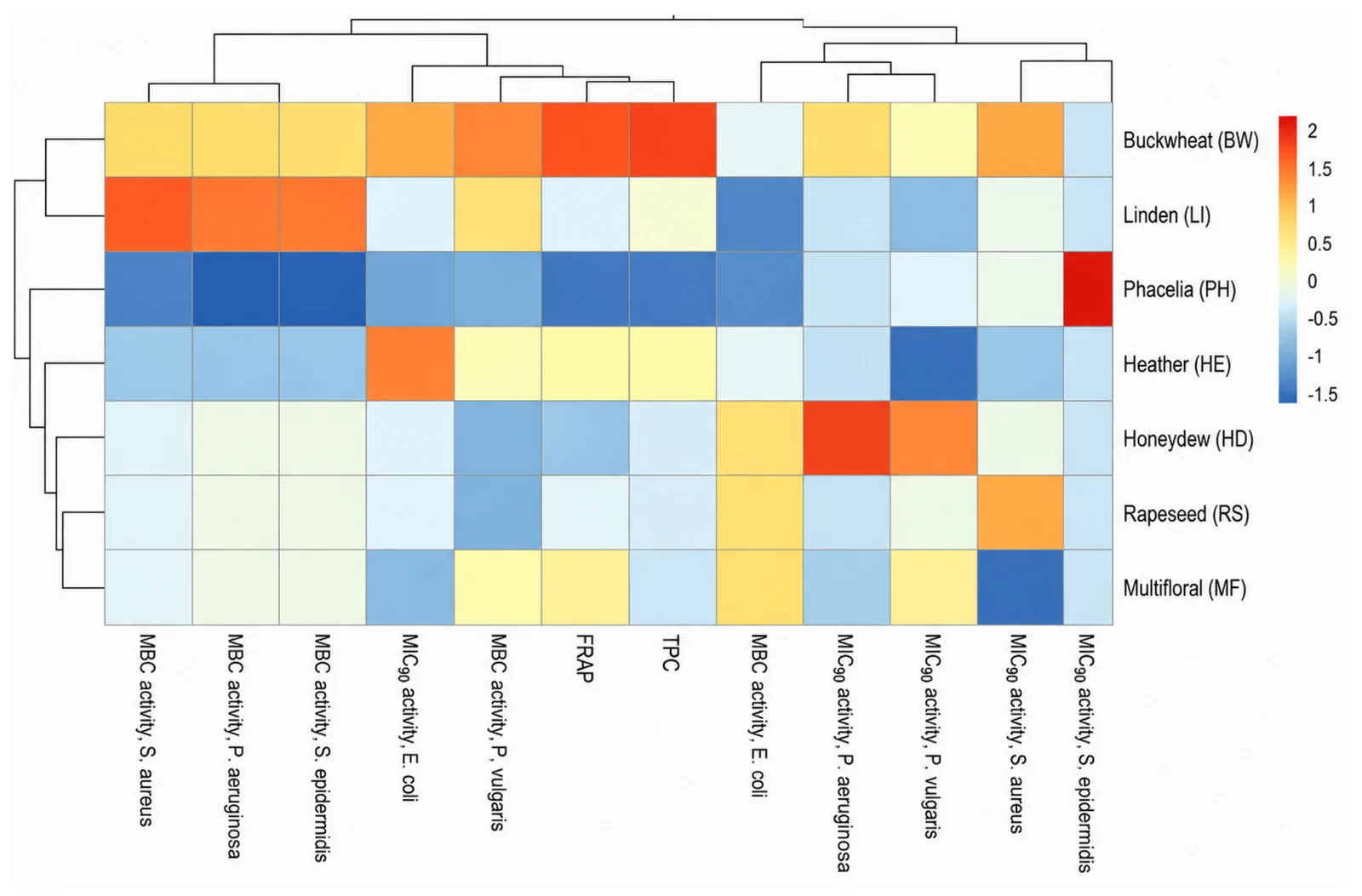
**Descriptive integrated bioactivity fingerprint of declared botanical honey types**. The heatmap displays standardised group-level profiles of species-specific MIC90 and MBC activity measures together with FRAP antioxidant activity and TPC. Warmer colours indicate values above the overall mean and cooler colours indicate values below the overall mean after standardisation. Dendrograms illustrate hierarchical similarity among the investigated declared botanical groups and bioactivity variables and are intended for descriptive visualisation only; they do not represent a validated botanical classification or prediction model. PH, phacelia; BW, buckwheat; LI, linden; RS, rapeseed; HD, honeydew; MF, multifloral; HE, heather.

These global indices represent descriptive summaries of the ordinal antimicrobial data and should not be interpreted as continuous quantitative measures of antibacterial potency. The integration of species-specific antimicrobial activity with FRAP and TPC further revealed heterogeneous multidimensional bioactivity patterns among the investigated declared botanical groups (Figure 9). Buckwheat honeys were characterised primarily by comparatively high FRAP and TPC values, whereas the remaining botanical groups displayed different combinations of inhibitory, bactericidal, and biochemical characteristics. The resulting bioactivity fingerprint is therefore presented as a descriptive multidimensional representation of the investigated samples rather than as a validated botanical classification or prediction tool.

## 3. Discussion

The present study supports the interpretation that honey antibacterial activity emerges from the interaction between the properties of the honey matrix and the susceptibility of the target bacterium. Within the investigated dataset, species-dependent differences in MIC90 and MBC were detected more consistently than differences among declared botanical types. This finding does not constitute a direct quantitative comparison of the relative contributions of bacterial susceptibility and botanical type because these factors were evaluated using different statistical frameworks. Rather, it indicates that antibacterial activity could not be consistently inferred from declared botanical classification alone. At the same time, integration of species-resolved antimicrobial responses with biochemical characteristics revealed botanical-type-associated multivariate structure that was not consistently apparent from individual endpoints. Together, these observations support a multidimensional interpretation of honey bioactivity in which botanical characteristics and microorganism-specific susceptibility contribute information at different analytical levels.

The biochemical results illustrate the substantial heterogeneity of honey even within a geographically restricted collection. Among the samples analysed here, buckwheat honeys exhibited the highest observed TPC and FRAP values. This is consistent with previous observations for Polish buckwheat honey and with studies showing that darker honeys frequently exhibit relatively high phenolic content and antioxidant capacity [11,12,15]. Importantly, however, botanical categories are not chemically homogeneous. In a recent analysis of 84 Polish varietal honeys, Jaśkiewicz et al. [11] identified clear botanical-type-associated differences in phenolic profiles and antioxidant activity, while multivariate analyses nevertheless showed incomplete separation among some honey varieties. This within-type heterogeneity is relevant to the present findings because biochemical variation associated with botanical source does not necessarily translate into a uniform biological response. Honey composition may additionally vary with geographical and environmental conditions, harvesting period, climatic factors and post-harvest handling [16,17,18,19,20]. Furthermore, TPC determined using the Folin–Ciocalteu assay should be regarded as an operational estimate of reducing phenolic-related constituents rather than as a compound-specific measurement of the complete phenolic composition.

The antimicrobial results further demonstrate why honey activity should not be assigned as a single characteristic of a botanical type. Declared botanical type only significantly differentiated MIC90 and MBC for *Staphylococcus aureus*, whereas no significant species-specific botanical-type effects were detected for the other four bacteria. Nevertheless, the significant *S. aureus* comparisons were substantial within the experimental concentration range. Rapeseed honeys reached the MIC90 endpoint at a median concentration of 25%, whereas the median multifloral sample did not reach 90% inhibition at the highest tested concentration of 50%. Similarly, the median operational MBC against *S. aureus* was 12.5% for linden honey and 50% for rapeseed honey. Thus, declared botanical type may be associated with marked differences in particular honey–bacterium combinations, but these effects cannot be generalised across microorganisms or antimicrobial endpoints. Earlier studies likewise reported pronounced variation in antimicrobial activity both among honey types and among samples assigned to the same floral category [8,21,22]. In Polish honeys, Grecka et al. [23] also observed substantial variation in MIC and MBC among samples and bacterial strains, including *S. aureus*, *S. epidermidis*, *E. coli* and *P. aeruginosa*, further illustrating that the antimicrobial phenotype depends on the specific honey–microorganism combination.

The repeated-measures analysis provides additional evidence for microorganism-specific susceptibility. *Proteus vulgaris* showed the lowest mean MBC code and was significantly more susceptible than several of the other species, while differences among bacterial species were even more evident for MIC90. Both *P. vulgaris* and *E. coli* exhibited lower mean MIC90 codes than several of the tested Gram-positive organisms and *P. aeruginosa*. These findings do not support a simple Gram-positive versus Gram-negative distinction in susceptibility to honey. Rather, they point to species-specific responses, which may reflect differences in cellular physiology, stress tolerance and sensitivity to the multiple antimicrobial components acting simultaneously within the honey matrix. Mundo et al. [6] similarly demonstrated that the same honey samples did not inhibit different foodborne microorganisms uniformly and that antibacterial activity could not be consistently assigned to a particular floral source. More recent investigations involving multiple honey types have likewise demonstrated microorganism-dependent variation in antibacterial response [8,10]. Consequently, comparison of honey antibacterial activity across studies requires consideration not only of honey composition and assay methodology but also of the bacterial species and strain used as the biological test system [24].

The relationship between the biochemical and antimicrobial dimensions of honey bioactivity was correspondingly complex. Three correlations reached nominal statistical significance: TPC was negatively associated with the *E. coli* MIC90 code, positively associated with the *E. coli* MBC code, and FRAP was positively associated with the *S. epidermidis* MIC90 code. However, none of the 20 biochemical–antimicrobial correlations remained statistically significant after Benjamini–Hochberg FDR correction. The opposite directions observed for inhibitory and bactericidal endpoints further argue against a simple model in which increasing TPC or reducing capacity directly translates into greater antibacterial potency. This contrasts with individual honey–bacterium systems in which stronger associations have been observed. For example, Núñez-Gómez et al. [12] reported relationships between TPC, antioxidant activity and antibacterial activity against *S. epidermidis* in Spanish honeys, while Alvarez-Suarez et al. [25] identified associations among compositional, antioxidant and antimicrobial characteristics in Cuban monofloral honeys. Conversely, broader analyses involving multiple honey samples and microorganisms have demonstrated substantially more heterogeneous relationships [8]. These differences are not necessarily contradictory; they indicate that the relationship between broad biochemical indices and antibacterial activity is context dependent. Moreover, the present study did not quantify individual phenolic compounds, hydrogen peroxide, methylglyoxal, antimicrobial peptides or other specific antibacterial constituents. The observed correlations should therefore be interpreted as exploratory associations rather than evidence for a direct biochemical mechanism.

The integrated multivariate analysis provides an important complementary perspective. When MIC90, MBC, FRAP and TPC were analysed jointly, declared botanical type was significantly associated with the overall bioactivity profile. Interpretation of the complete-dataset PERMANOVA nevertheless requires caution because multivariate dispersion also differed significantly among botanical groups. PERMANOVA may respond both to differences in group centroids and to heterogeneity of within-group dispersion; consequently, the significant result obtained from the complete dataset cannot be attributed exclusively to differences in central multivariate profiles. The sensitivity analysis excluding phacelia and honeydew, which were each represented by a single sample, is therefore particularly informative. The association between declared botanical type and integrated bioactivity remained significant, whereas multivariate dispersion no longer differed among groups. This reduced analysis provides a more interpretable basis for the botanical-type-associated multivariate pattern observed in the adequately represented groups. Similar studies demonstrate the value of integrating multiple dimensions of honey composition and biological activity: Alcoléa et al. [13] used the multivariate analysis of biochemical, antioxidant and antimicrobial characteristics to identify the structures associated with botanical and geographical origin, while polyparametric approaches have likewise shown that information distributed across several honey characteristics can provide greater differentiation than individual measurements considered separately [14].

The bioactivity fingerprint developed in the present study should therefore be understood as an integrative descriptive representation rather than a botanical authentication tool. Species-specific MIC90 and MBC responses contribute dimensions that are not captured by TPC or FRAP alone, while biochemical traits provide complementary information that is not evident from antimicrobial endpoints considered separately. Importantly, the global MIC90 and MBC activity indices were constructed by equally weighting all five bacterial species after ordinal rescaling. Because MIC90 and MBC originated from serial dilution categories, and because values exceeding 50% were grouped into the highest right-censored category, the resulting 0–1 scores do not constitute continuous measurements of antimicrobial potency. They provide a common descriptive scale that facilitates integration and visual comparison. Likewise, the hierarchical clustering and ordination patterns identify similarity within the present dataset but should not be interpreted as validated prediction or classification performance. Independent validation using substantially larger collections would be required before such a profile could be considered for botanical discrimination.

Several limitations are important for interpreting the botanical-type-associated component of the study. The botanical classification was based on beekeeper declaration according to the dominant floral resource, harvest period and apiary location and was not independently authenticated by melissopalynological or chemical methods. Consequently, uncertainty in botanical assignment may contribute to within-group heterogeneity and potentially attenuate detectable differences among declared botanical types. The terminology “declared botanical type” is therefore particularly important when interpreting the present findings. Group sizes were also markedly unequal, with phacelia and honeydew represented by only one sample each and buckwheat and heather by two samples. Results for these sparsely represented categories are descriptive and cannot be regarded as estimates of the typical properties of the corresponding honey types. The sensitivity analysis excluding singleton groups mitigates this issue for the integrated multivariate analysis but does not eliminate the imbalance of the overall study design.

The MBC procedure represents an additional methodological limitation. Honey solutions were filter-sterilised and uninoculated honey controls confirmed the absence of microbial contamination; however, no neutralisation or washing step was performed before direct transfer of 10 µL aliquots from honey-containing wells onto MHA. Residual antimicrobial constituents transferred with the aliquot may therefore have continued to act on the agar surface and could potentially have lowered the apparent operational MBC. This possibility does not invalidate the observed bactericidal patterns but means that the MBC values should be interpreted specifically within the operational conditions of the assay. Finally, biochemical characterisation was restricted to TPC and FRAP, and antibacterial testing involved five ATCC reference strains. Future studies incorporating independently authenticated botanical origin, balanced sampling, detailed chemical profiling and a broader panel of reference and clinical isolates will be necessary to determine how reproducible the multidimensional patterns identified here are across honey collections and microbial targets.

Overall, the findings indicate that neither botanical classification, biochemical characteristics nor microorganism susceptibility alone provides a complete description of honey antibacterial bioactivity. Species-dependent differences were evident across the antimicrobial assays, while declared botanical type contributed a detectable structure primarily at selected individual endpoints and within the integrated multivariate profile. The principal contribution of the present study is therefore not the establishment of a hierarchy between these sources of variation, but the demonstration that their integration provides a more informative description of honey bioactivity than the interpretation of any single dimension in isolation.

## 4. Materials and Methods

### 4.1. Honey Samples

A total of 37 honey samples representing seven declared botanical types were included in the study: phacelia (PH; n = 1), buckwheat (BW; n = 2), linden (LI; n = 4), rapeseed (RS; n = 12), honeydew (HD; n = 1), multifloral (MF; n = 15), and heather honey (HE; n = 2). The samples were obtained from apiaries located in the West Pomeranian region of Poland during the 2026 beekeeping season. Botanical type was assigned by the participating beekeepers based on the dominant floral resource available to the colonies, the harvesting period, and the location of the apiary. These data were recorded by the beekeepers in a dedicated sampling form accompanying each honey sample. Botanical classification was therefore treated throughout the study as a declared botanical type and was not independently verified by melissopalynological analysis or chemical authentication. Each analysed honey sample represented a separately collected biological sample. Only one sample of a given declared botanical type was collected from an individual hive. Where more than one sample of the same botanical type originated from the same apiary, the samples were obtained from different hives and were treated as separate biological samples.

Following collection, the samples were transferred to sterile, airtight containers and stored in the dark at 18 °C until analysis to minimise changes in their physicochemical and biological properties. All laboratory analyses were performed within 2 weeks of sample collection.

### 4.2. Bacterial Strains and Culture Conditions

Five reference bacterial strains were used to assess the antibacterial activity of the honey samples: *Staphylococcus epidermidis* ATCC 12228, *Escherichia coli* ATCC 25922, *Staphylococcus aureus* ATCC 25923, *Pseudomonas aeruginosa* ATCC 15442, and *Proteus vulgaris* ATCC 29905. All strains were obtained from the American Type Culture Collection (ATCC, Manassas, VA, USA).

Before each antibacterial susceptibility assay, bacterial strains were cultured on Mueller–Hinton agar (MHA; Oxoid Ltd., Basingstoke, UK) under aerobic conditions at 37 °C for 18–24 h. Fresh bacterial colonies were subsequently suspended in sterile phosphate-buffered saline (PBS, pH 7.4; Oxoid Ltd., Basingstoke, UK), and the turbidity of each suspension was adjusted to 0.5 McFarland using a DEN-1B densitometer (Biosan, Riga, Latvia).

The standardised bacterial suspensions were subsequently diluted in Mueller–Hinton broth (MHB; Oxoid Ltd., Basingstoke, UK) to obtain the working inoculum used for broth microdilution assays. The target final bacterial concentration in the assay was approximately 5 × 10^5^ CFU/mL. The inoculum concentration was experimentally verified by viable-count plating and enumeration of colony-forming units.

### 4.3. Determination of the Operational MIC90

The inhibitory activity of each honey sample was determined using a broth microdilution approach adapted from the Clinical and Laboratory Standards Institute (CLSI) M07 methodology for antimicrobial susceptibility testing of aerobically growing bacteria [26]. Because bacterial growth was quantified spectrophotometrically, the inhibitory endpoint used in this study was operationally defined as MIC90, i.e., the lowest tested honey concentration producing at least 90% inhibition of bacterial growth relative to the untreated growth control.

Honey solutions were prepared in double-strength Mueller–Hinton broth (2× MHB) and sterilised by filtration through 0.22 µm PTFE membrane filters. Two-fold serial dilutions were prepared in sterile 96-well microtitre plates. For each assay well, 100 µL of the appropriate honey dilution was combined with 100 µL of bacterial inoculum, giving a final assay volume of 200 µL. The final honey concentrations were 50%, 25%, 12.5%, 6.25%, 3.125%, 1.56%, 0.78%, and 0.39% (*v*/*v*). The target final bacterial concentration was approximately 5 × 10^5^ CFU/mL and was experimentally verified by viable-count plating and enumeration of colony-forming units.

Each plate included a growth control containing bacterial inoculum in Mueller–Hinton broth without honey and a sterility control containing uninoculated medium. For each honey concentration, an uninoculated honey-background control containing the corresponding honey dilution in Mueller–Hinton broth was included to account for the intrinsic absorbance and turbidity of the honey matrix. Background normalisation was performed by subtracting the absorbance of the corresponding uninoculated honey-medium control from the absorbance of the inoculated honey-containing well. No negative background-corrected absorbance values were obtained. Microplates were incubated aerobically at 37 °C for 24 h, after which bacterial growth was assessed by measuring absorbance at 600 nm using a Multiskan FC microplate reader (Thermo Fisher Scientific, Waltham, MA, USA). Growth inhibition was calculated relative to the untreated bacterial growth control according to the following equation:

Growth inhibition (%) = [1 − (OD_600_ background-corrected sample/OD_600_ growth control)] × 100.

The operational MIC90 was defined as the lowest tested honey concentration resulting in ≥90% inhibition of bacterial growth relative to the untreated growth control. The ≥90% inhibition threshold was selected as an objective spectrophotometric endpoint consistent with previously reported honey microdilution assays [5], while retaining the serial-dilution design used in the present study. Accordingly, MIC90 should be regarded as an operational absorbance-based endpoint rather than as the conventional visually determined MIC. When the ≥90% inhibition threshold was not reached at the highest honey concentration tested, the MIC90 was recorded as >50% (*v*/*v*).

MIC90 determinations were performed in three independent experiments for each honey sample–bacterial species combination. The median MIC90 obtained across the three independent experiments was used as the representative value for subsequent statistical analyses.

### 4.4. Determination of Minimum Bactericidal Concentration

Minimum bactericidal concentration (MBC) was determined as a culture-based viability endpoint using a subculture approach adapted from the CLSI M26 guidance for bactericidal activity testing [27]. MBC assessment was performed using aliquots from the same broth microdilution plates used for determination of the inhibitory endpoint.

Following 24 h incubation of the microdilution plates, 10 µL aliquots from four predefined honey concentrations (6.25%, 12.5%, 25%, and 50% *v*/*v*) were spot-inoculated onto Mueller–Hinton agar (MHA) plates. The agar plates were incubated aerobically at 37 °C for 24 h and subsequently examined for bacterial colony growth.

The operational MBC was defined as the lowest of the four tested honey concentrations from which no visible bacterial colonies were recovered following subculture. Any visible colony growth was considered evidence of bacterial recovery, indicating that the MBC endpoint had not been reached at that concentration. When no colonies were recovered from the lowest concentration evaluated for MBC, 6.25% (*v*/*v*), the result was recorded as ≤6.25% (*v*/*v*). When bacterial growth was recovered from all four concentrations, including 50% (*v*/*v*) honey, the MBC was recorded as >50% (*v*/*v*). MBC assessment was therefore not conditional on the spectrophotometric MIC90 endpoint being reached within the tested concentration range.

Honey solutions used in the antimicrobial assays had been sterilised by filtration through 0.22 µm membrane filters, and uninoculated honey controls were included to confirm the absence of microbial contamination. No neutralisation or washing step was applied before transfer of the 10 µL aliquots onto MHA, and no dedicated antimicrobial carry-over control was performed during the MBC assay.

MBC determinations were performed in three independent experiments for each honey sample–bacterial species combination. The median MBC obtained across the three independent experiments was used as the representative value for subsequent statistical analyses. Growth and sterility controls were processed in parallel to confirm bacterial viability and medium sterility, respectively.

### 4.5. Preparation of Honey Extracts for Biochemical Analyses

Methanolic extracts of honey were prepared for the determination of total phenolic content (TPC) and antioxidant activity using the FRAP assay.

For each honey sample, three independent extracts were prepared from separate 1.0 g aliquots of the same sample. Each aliquot was transferred to a 10 mL volumetric flask and extracted with 3.0 mL of analytical-grade methanol (Sigma-Aldrich, St. Louis, MO, USA). Samples were shaken for 20 min at 130 rpm using an orbital shaker and subsequently centrifuged at 10,000 rpm for 10 min at 4 °C using a refrigerated centrifuge.

For each independent extract, the extraction procedure was performed three times sequentially on the same honey aliquot, using 3.0 mL of methanol at each extraction step. Following centrifugation, the supernatant was carefully collected after each extraction. The three successive supernatants were pooled, and the combined extract was adjusted to a final volume of 10.0 mL with methanol. The resulting methanolic extracts were used for TPC and FRAP determinations. No separate extraction-recovery validation was performed.

### 4.6. Determination of Total Phenolic Content

Total phenolic content (TPC) was determined using the Folin–Ciocalteu colourimetric method based on the procedure described by Singleton and Rossi [28], with modifications. For each determination, 25 µL of Folin–Ciocalteu reagent and 25 µL of ultrapure water were added to a well of a 96-well microplate, followed by 10 µL of the honey extract. The mixture was incubated for 5 min, after which 40 µL of 7.5% (*w*/*v*) sodium carbonate solution was added. Following mixing, 100 µL of ultrapure water was added to obtain a final reaction volume of 200 µL per well. The reaction mixture was incubated for 30 min at room temperature.

Absorbance was measured at 725 nm using a Multiskan FC microplate reader (Thermo Fisher Scientific, Waltham, MA, USA). A calibration curve was prepared using chlorogenic acid over the concentration range of 0–500 µg/mL. The calibration equation was y = 0.0022x + 0.0127, with a coefficient of determination of R^2^ = 0.9995. TPC was calculated from the calibration equation, considering the amount of honey used for extract preparation, and expressed as milligrams of chlorogenic acid equivalents per kilogram of honey (mg CAE/kg honey).

The Folin–Ciocalteu assay was used as an operational estimate of total phenolic content. Because the reagent responds to reducing substances in addition to phenolic compounds, the resulting TPC values should not be interpreted as a direct quantitative measurement of all individual phenolic constituents present in honey.

Three independently prepared extracts were analysed for each honey sample.

### 4.7. Determination of Antioxidant Activity by the FRAP Assay

Antioxidant activity was determined using the ferric-reducing antioxidant power (FRAP) assay based on the method originally described by Benzie and Strain [29], with modifications. The FRAP reagent was freshly prepared before each assay by mixing 10 mmol/L TPTZ solution prepared in 40 mmol/L HCl, 20 mmol/L FeCl_3_ solution, and 0.3 mol/L acetate buffer (pH 3.6) at a volume ratio of 1:1:10.

For each determination, 2.0 mL of freshly prepared FRAP reagent was mixed with 0.2 mL of the honey extract. Samples were incubated at 37 °C for 30 min, after which absorbance was measured at 593 nm.

A calibration curve was prepared using Trolox over the concentration range of 0–500 µg/mL. The calibration equation was y = 0.0030x − 0.0007, with a coefficient of determination of R^2^ = 0.9998. FRAP antioxidant activity was calculated from the Trolox calibration curve and expressed as µg/mL Trolox equivalents (TE).

Three independently prepared extracts were analysed for each honey sample.

### 4.8. Statistical Analysis

Statistical analyses were performed using R statistical software (R Foundation for Statistical Computing, Vienna, Austria; version 4.6.1). A total of 37 honey samples and five bacterial species were included in the antimicrobial dataset, corresponding to 185 honey sample-bacterial species combinations for both MIC90 and MBC assessments. The median MIC90 and MBC values obtained from three independent experiments were used as representative sample-level values for subsequent analyses.

MIC90 and MBC values were treated as ordered antimicrobial activity measurements because they were derived from serial dilution steps, with lower concentrations indicating greater antimicrobial activity. Values exceeding the highest tested honey concentration (>50% *v*/*v*) were treated as right-censored observations and assigned to the highest ordinal category. This approach preserved the ordering of the observations but did not distinguish among true endpoint values above the upper experimental limit. Consequently, observations within the >50% category should not be interpreted as having identical true MIC90 or MBC values, and compression of variation at the upper end of the scale may reduce discrimination among less susceptible honey–bacterium combinations.

Continuous biochemical parameters were summarised using the mean, median, and range. Owing to unequal botanical-group sizes and the ordinal, non-normally distributed nature of the antimicrobial endpoints, non-parametric statistical methods were used for comparisons of antimicrobial activity.

For each bacterial species separately, differences in MIC90 and MBC among declared botanical honey types were assessed using the Kruskal–Wallis test. Where an overall test was statistically significant, pairwise comparisons were performed using Dunn’s post hoc test. *p*-values from multiple pairwise comparisons were adjusted using the Benjamini–Hochberg false discovery rate (FDR) procedure.

Differences in susceptibility among the five bacterial species were evaluated using the Friedman test, with each honey sample treated as a repeated-measures block. When the Friedman test was significant, pairwise comparisons between bacterial species were performed using paired Wilcoxon signed-rank tests, with Benjamini–Hochberg adjustment for multiple comparisons. The bacterial species and declared botanical-type analyses addressed different statistical questions and were not used to estimate or directly compare the relative effect sizes of these two factors within a common statistical model.

Associations between biochemical characteristics and antimicrobial activity were explored using Spearman’s rank correlation coefficient (ρ). TPC and FRAP antioxidant activity were correlated separately with species-specific MIC90 and MBC ordinal codes, resulting in 20 biochemical–antimicrobial correlations. Because these analyses were exploratory, nominal *p*-values were examined to describe the direction and strength of potential associations. In response to the multiplicity of comparisons, Benjamini–Hochberg FDR-adjusted *p*-values were additionally calculated as a sensitivity analysis. The correlation analyses were considered exploratory and were not interpreted as confirmatory evidence of mechanistic relationships.

Separate PERMANOVA analyses were performed for the five-variable MIC90 and five-variable MBC profiles to assess whether declared botanical type was associated with the multivariate inhibitory or bactericidal activity profiles independently of the biochemical parameters. Homogeneity of multivariate dispersion was evaluated using the betadisper procedure for each analysis.

To evaluate differences in the integrated bioactivity profile among declared botanical honey types, permutational multivariate analysis of variance (PERMANOVA) was performed using the adonis2 function implemented in the vegan package in R. The integrated dataset comprised five species-specific MIC90 variables, five species-specific MBC variables, FRAP antioxidant activity, and TPC. Because these variables were expressed on different scales, each variable was z-standardised to a mean of zero and a standard deviation of one before calculation of Euclidean distances. PERMANOVA was performed using 9999 permutations, with declared botanical type as the explanatory factor.

Differences in within-group multivariate dispersion were assessed using the betadisper procedure followed by a permutation test. Because PERMANOVA can be influenced by differences in within-group dispersion as well as differences in group centroids, significant PERMANOVA results were interpreted in conjunction with the corresponding dispersion test. The primary analysis included all seven declared botanical types. Because phacelia and honeydew were each represented by a single sample, an additional sensitivity analysis was performed after exclusion of these singleton groups. This analysis allowed the botanical-type association to be evaluated in a dataset in which within-group dispersion could be meaningfully assessed for all included groups.

Pairwise PERMANOVA comparisons were performed only between declared botanical types represented by more than one sample. The *p*-values obtained from pairwise comparisons were adjusted for multiple testing using the Benjamini–Hochberg FDR procedure.

The multivariate structure of the integrated bioactivity dataset was visualised using principal coordinates analysis (PCoA) based on Euclidean distances. PCoA was performed for the complete dataset and—as a sensitivity visualisation—after exclusion of declared botanical types, represented by a single sample. Associations of individual antimicrobial and biochemical variables with the ordination configuration of the complete dataset were assessed using the envfit function with 9999 permutations. The *p*-values obtained for fitted variables were adjusted using the Benjamini–Hochberg procedure. A complementary constrained ordination was performed using constrained analysis of principal coordinates (CAP), equivalent to distance-based redundancy analysis (dbRDA), based on Euclidean distances, with declared botanical type used as the constraining factor.

For descriptive integration and visualisation of antimicrobial activity, MIC90 and MBC ordinal codes were transformed separately to a common 0–1 activity scale. For each endpoint, the direction of the ordinal scale was reversed and rescaled according to

activity score = (Cmax − C)/(Cmax − Cmin),

where *C* represents the ordinal MIC90 or MBC code for a given honey–bacterium combination and *Cmin* and *Cmax* represent the lowest and highest possible ordinal codes, respectively. Thus, a score of 1 represented the strongest activity category and a score of 0 represented the weakest activity category. The transformation preserved the rank ordering of the dilution-based endpoints but should be regarded solely as an ordinal descriptive rescaling; it does not imply that equal numerical differences on the 0–1 scale represent equal quantitative differences in antimicrobial potency.

Species-specific activity scores were subsequently aggregated across the five bacterial species to generate global MIC90 and global MBC activity indices for each honey sample. Each bacterial species contributed equally to the corresponding global index, which was calculated as the arithmetic mean of the five species-specific activity scores. These indices were used exclusively as descriptive measures for integrated visualisation and were not treated as validated quantitative measures of overall antibacterial potency or as predictive classification tools.

All statistical tests were two-sided. A *p*-value < 0.05 was considered statistically significant. Where correction for multiple comparisons was applied, Benjamini–Hochberg-adjusted *p*-values (p_BH) are reported.

## 5. Conclusions

The present study supports the interpretation of honey antibacterial activity as a multidimensional phenotype shaped by both the properties of the honey matrix and microorganism-specific susceptibility. Within the investigated dataset, species-dependent differences in MIC90 and MBC were detected more consistently than differences among declared botanical types. This observation should not be interpreted as a direct quantitative comparison of the relative contributions of bacterial susceptibility and botanical type but rather indicates that declared botanical classification was not consistently associated with inhibitory or bactericidal responses across all bacterial species examined.

At the same time, the limited and directionally inconsistent relationships between total phenolic content, FRAP antioxidant activity, and MIC90/MBC indicate that no single biochemical parameter adequately captures the antibacterial phenotype of honey. Importantly, the integration of species-resolved MIC90 and MBC profiles with FRAP antioxidant activity and total phenolic content revealed significant botanical-type-associated structuring of overall bioactivity. This association remained significant after the exclusion of botanical groups represented by single samples, while differences in multivariate dispersion were no longer detected, providing additional support for the botanical-type-associated pattern observed in the integrated dataset.

The resulting bioactivity fingerprint provides an integrative description of how inhibitory, bactericidal, and biochemical characteristics coexist within the investigated declared botanical honey types, including patterns that were not consistently apparent when individual endpoints were considered separately. The bioactivity fingerprint proposed here should, however, be regarded as a descriptive multidimensional framework rather than a validated botanical classification or prediction tool.

Botanical classification was based on beekeeper declaration and was not independently authenticated, group sizes were unequal, biochemical characterisation was restricted to TPC and FRAP, and antibacterial activity was assessed using a limited panel of reference bacterial strains. Consequently, the identified patterns should not be generalised beyond the honey samples and bacterial species investigated here without further validation. Nevertheless, the present findings support the use of integrated, species-resolved microbiological and biochemical profiling as a complementary approach for describing the multidimensional bioactivity of honey.

## Data Availability

The data supporting the findings of this study are included within the article and its Supplementary Materials. The raw data supporting the conclusions of this article are available from the corresponding author upon reasonable request.

## Supplementary Materials

The following supporting information can be downloaded at: https://www.mdpi.com/article/10.3390/molecules31173048/s1. Table S1: Numerical summary of biochemical and antimicrobial characteristics according to declared botanical honey type; Table S2: Exploratory Spearman rank correlations between biochemical characteristics and species-specific antimicrobial endpoints, including Benjamini-Hochberg FDR-adjusted *p*-values.
