# Supplementary material for: Species-Dependent Antibacterial Responses and Bioactivity Patterns Associated with Declared Botanical Type in Honey: An Integrated Biochemical and Microbiological Analysis
Source: Molecules. 2026 Aug 30;31(17):3048. doi: 10.3390/molecules31173048 (PMC13567264; doi:10.3390/molecules31173048)
Supplement: Supplementary file 1 [file molecules-31-03048-s001.zip › molecules-4512557-supplementary.pdf]

Species-dependent antibacterial responses and bioactivity patterns associated with declared botanical type in honey: an integrated biochemical and microbiological analysis.

SUPPLEMENTARY MATERIALS

Table S1. Numerical summary of biochemical and antimicrobial characteristics according to declared botanical honey type

Table S1A. Biochemical characteristics

Declared botanical type	Abbreviation	n	FRAP, mean (µg/mL TE)	TPC, mean (mg CAE/kg honey)
Phacelia	PH	1	92.57	748.80
Buckwheat	BW	2	195.90	1320.82
Linden	LI	4	131.46	1287.77
Rapeseed	RS	12	123.47	937.08
Honeydew	HD	1	112.57	935.67
Multifloral	MF	15	147.81	934.22
Heather	HE	2	151.24	1061.02

Table S1B. Species-specific MIC90 and MBC profiles

Declared botanical type	*E. coli* MIC90	*E. coli* MBC	*P. aeruginosa* MIC90	*P. aeruginosa* MBC	*P. vulgaris* MIC90	*P. vulgaris* MBC
PH	8 [8-8]	5 [5-5]	8 [8-8]	5 [5-5]	7 [7-7]	4 [4-4]
BW	5.0 [3-7]	4.5 [4-5]	5.5 [3-8]	3.5 [3-4]	6.0 [4-8]	2.0 [1-3]
LI	7.0 [1-8]	5.0 [3-5]	8.0 [7-8]	3.0 [3-4]	7.5 [4-8]	2.5 [1-4]
RS	7.0 [1-9]	4.0 [3-5]	8.0 [7-9]	4.0 [3-5]	6.5 [3-9]	4.0 [3-5]
HD	7 [7-7]	4 [4-4]	3 [3-3]	4 [4-4]	4 [4-4]	4 [4-4]
MF	8 [1-9]	4 [2-5]	8 [3-9]	4 [3-5]	7 [3-9]	3 [1-5]
HE	4.5 [1-8]	4.5 [4-5]	8.0 [8-8]	4.5 [4-5]	9.0 [9-9]	3.0 [2-4]

Declared botanical type	*S. aureus* MIC90	*S. aureus* MBC	*S. epidermidis* MIC90	*S. epidermidis* MBC
PH	8 [8-8]	5 [5-5]	8 [8-8]	5 [5-5]
BW	7.0 [7-7]	3.0 [2-4]	9.0 [9-9]	3.5 [3-4]
LI	8.0 [7-9]	2.0 [1-2]	8.5 [8-9]	3.0 [3-3]
RS	7.0 [5-9]	4.0 [3-5]	9.0 [1-9]	4.0 [2-5]
HD	8 [8-8]	4 [4-4]	9 [9-9]	4 [4-4]
MF	9 [6-9]	4 [2-5]	9 [1-9]	4 [1-5]
HE	8.5 [8-9]	4.5 [4-5]	9.0 [9-9]	4.5 [4-5]

Abbreviations: PH, phacelia; BW, buckwheat; LI, linden; RS, rapeseed; HD, honeydew; MF, multifloral; HE, heather; FRAP, ferric reducing antioxidant power; TPC, total phenolic content; MIC90, operational minimum inhibitory concentration producing ≥90% growth inhibition; MBC, minimum bactericidal concentration. MIC90 and MBC values are presented as median ordinal code [range]. MIC90 codes correspond to the following concentration categories: 1 = 0.39%, 2 = 0.78%, 3 = 1.56%, 4 = 3.125%, 5 = 6.25%, 6 = 12.5%, 7 = 25%, 8 = 50%, and 9 = >50% (v/v). MBC codes correspond to: 1 = ≤6.25%, 2 = 12.5%, 3 = 25%, 4 = 50%, and 5 = >50% (v/v). Lower ordinal codes indicate stronger antimicrobial activity. Values for botanical types represented by a single sample (PH and HD) are descriptive only.

Table S2. Exploratory Spearman rank correlations between biochemical characteristics and species-specific antimicrobial endpoints, including Benjamini–Hochberg FDR-adjusted p-values.

Biochemical parameter	Bacterial species	Endpoint	Spearman ρ	Nominal p-value	p_BH
**TPC**	*Escherichia coli*	MIC90	-0.390	0.0195	0.2873
**TPC**	*Escherichia coli*	MBC	0.360	0.0298	0.2873
**FRAP**	*Staphylococcus epidermidis*	MIC90	0.350	0.0431	0.2873
**FRAP**	*Proteus vulgaris*	MIC90	-0.270	0.1170	0.5850
**TPC**	*Proteus vulgaris*	MBC	-0.230	0.1690	0.6367
**FRAP**	*Proteus vulgaris*	MBC	-0.230	0.1910	0.6367
**FRAP**	*Staphylococcus epidermidis*	MBC	-0.210	0.2250	0.6429
**FRAP**	*Pseudomonas aeruginosa*	MBC	-0.170	0.3200	0.7369
**FRAP**	*Staphylococcus aureus*	MBC	-0.160	0.3670	0.7369
**TPC**	*Proteus vulgaris*	MIC90	0.150	0.3900	0.7369
**FRAP**	*Escherichia coli*	MBC	-0.140	0.4250	0.7369
**TPC**	*Staphylococcus aureus*	MBC	-0.130	0.4450	0.7369
**TPC**	*Pseudomonas aeruginosa*	MIC90	-0.120	0.4790	0.7369
**TPC**	*Staphylococcus aureus*	MIC90	-0.080	0.6490	0.8565
**FRAP**	*Escherichia coli*	MIC90	0.081	0.6490	0.8565
**TPC**	*Staphylococcus epidermidis*	MIC90	0.064	0.7170	0.8565
**FRAP**	*Pseudomonas aeruginosa*	MIC90	0.062	0.7280	0.8565
**TPC**	*Staphylococcus epidermidis*	MBC	-0.044	0.7990	0.8790
**TPC**	*Pseudomonas aeruginosa*	MBC	-0.029	0.8670	0.8790
**FRAP**	*Staphylococcus aureus*	MIC90	0.027	0.8790	0.8790

Abbreviations: TPC, total phenolic content; FRAP, ferric reducing antioxidant power; MIC90, operational minimum inhibitory concentration producing ≥90% growth inhibition; MBC, minimum bactericidal concentration; ϱ, Spearman's rank correlation coefficient; p_BH, p-value adjusted using the Benjamini-Hochberg false discovery rate (FDR) procedure. Nominal p-values are reported for the exploratory correlation analysis, whereas p_BH values were calculated across all 20 biochemical-antimicrobial correlations. Lower MIC90 and MBC ordinal codes indicate stronger antimicrobial activity. Accordingly, negative ϱ values indicate an association between higher TPC or FRAP values and stronger antimicrobial activity, whereas positive ϱ values indicate an association with weaker antimicrobial activity. None of the correlations remained statistically significant after FDR correction (all $p\_BH > 0.05$).
